# Knowledge About the Importance of Early Diagnosis and Treatment of Oral Potentially Malignant Disorders Among the South Indian Population: An Institutional Retrospective Study

**DOI:** 10.7759/cureus.57740

**Published:** 2024-04-06

**Authors:** Suvarna Kizhakkoottu, Pratibha Ramani

**Affiliations:** 1 Oral Pathology and Microbiology, Saveetha Dental College and Hospitals, Saveetha Institute of Medical and Technical Sciences, Saveetha University, Chennai, IND

**Keywords:** referral, opmd, oral potentially malignant disorders, early diagnosis, awareness, knowledge

## Abstract

Background: The significant malignant transformation rates of oral potentially malignant disorders (OPMDs) demand early diagnosis and proper management of OPMDs not only to reduce symptoms but also to prevent their aggressive outcomes. This retrospective study aimed to quantify the need for patient-related awareness in identifying OPMDs by quantitatively evaluating the association between the type of referral in OPMD cases. This study also aims to analyze the association between gender and types of referral in OPMDs.

Materials and methods: The sample size of n=1577 (500-leukoplakia, 500-oral submucous fibrosis (OSMF), 500-lichen planus, 77-lichenoid reaction) was considered in the present retrospective study. Data regarding the sample cases were extracted from the common patient database of the Saveetha Dental College and Hospitals from June 2019 to February 2024. Random sampling method was used, and the OPMDs were categorized into two groups based on the chief complaint as self-referred and specialist-referred cases. The segregated data were tabulated in Microsoft Excel (Microsoft® Corp., Redmond, WA) and then exported to IBM SPSS Statistics for Windows, Version 23 (Released 2015; IBM Corp., Armonk, New York, United States) for statistical analysis. Pearson's chi-square test was conducted to analyze the association of referral type, OPMDs, and gender.

Results: Out of 1577 OPMD cases, 929 (58.9%) were specialist-referral cases and 648 (41.1%) were self-referral cases. Among OPMDs, lichen planus was the most self-referred 310 (62%) and leukoplakia was the most specialist-referred 470 (78.6%) category. This study found a statistically significant correlation between the type of referrals and the type of OPMDs (p=0.000). Self-referral was more commonly observed in females (23.3%) than males (17.8%) in general and among all categories of OPMDs except lichenoid reactions. This observation was also statistically significant (p=0.000).

Conclusion: Among OPMDs selected in the present study, lichen planus and OSMF were more self-referred and leukoplakia cases were mostly specialist-referred. This study highlights the need of detecting less symptomatic lesions, such as leukoplakia, which has a high risk of malignant transformation. The lack of awareness about the identification of OPMDs among patients can result in delayed diagnosis and treatment, which may further result in progression to aggressive outcomes.

## Introduction

Oral cancer is a growing concern in the medical community, comprising 2-4% of all cases worldwide [1]. More than 90% of oral cancer cases that have been documented worldwide are oral squamous cell carcinomas, which affect over 300,000 individuals annually [2]. Clinically evident mucosal lesions that may be precancerous such as erythroplakia, leukoplakia, oral submucous fibrosis (OSMF), and lichen planus can occur before the development of oral cancer [3]. The term oral potentially malignant disorders (OPMDs) was proposed by the WHO in 2005 and this includes potentially malignant lesions and potentially malignant conditions. The spectrum of OPMDs includes mainly oral leukoplakia, erythroplakia, erythroleukoplakia, OSMF, palatal lesions in reverse smokers, oral lichen planus, and oral lichenoid reactions [4]. Under specific altered tissue microenvironments, OPMDs have the potential to grow and progress to aggressive oral cancer within a given time frame [5]. 

The sequence of malignant transformation of OPMDs depends on the progression of epithelial dysplasia and variations in the connective tissue tumor microenvironment. This transformation does not always follow a predictable sequence from mild to moderate to severe dysplasia and dysplastic cases may have the ability to revert to normal mucosa [6]. The malignant transformation of OPMDs is dependent upon several factors, which may be related to the lesion or the patient [7]. A systematic review by Warnakulasuriya and Ariyawardana stated that the overall malignant transformation rate of leukoplakia is 1.5% to 34%, which is 3% in homogenous lesions and 14.5% in non-homogeneous lesions [8]. The overall malignant transformation rate and annual transformation rate of OSMF have been reported as 4.2% and 0.73%, respectively [9]. The malignant transformation rate of oral lichen planus and lichenoid lesions is 2.28% and 1.95%, respectively [10]. The malignant transformation of OPMDs can be strongly associated with factors like the site, morphology of the lesion, and habits [7]. 

The significant malignant transformation rate in different potentially malignant diseases demands early diagnosis and proper management of these lesions. The early screening and diagnosis of OPMDs not only reduce the symptoms but also prevent its malignant transformation to oral squamous cell carcinoma [11]. Despite the mass screening programs, the basic awareness regarding OPMDs and the probability of malignant transformation in those lesions among the general population will improve the early diagnosis and treatment of OPMDs. This retrospective study aimed to evaluate the type of referral among the reported OPMD cases, thereby quantifying the need for patient-related awareness in identifying OPMDs. The present study also stresses the need for the early diagnosis of OPMDs and its treatment before malignant transformation leading to poor prognosis.

## Materials and methods

The current study was carried out with permission from Saveetha Dental College and Hospitals, Chennai. This study was approved ethically under the Institutional Human Ethical Committee with ethical number SDC/SIHEC/2020/DIASDATA/0619-0320. The study design of the present study was retrospective and the duration of conduct of the present study was from 2019 to 2024. In this study, we analyzed the case sheets of all the patients reported to the Department of Oral Medicine, Saveetha Dental College and Hospitals. We reviewed and analyzed the case records of patients who visited our institution between June 2019 and February 2024. Initially, we segregated the OPMD cases and further categorized them into different categories like oral lichen planus, OSMF, lichenoid reactions, and leukoplakia. Only those cases that had proper patient follow-up data, diagnosis, clinical details, and treatment data were included. Reactive lesions and lesions of questionable diagnosis were excluded from the present study. The screening process based on the inclusion and exclusion criteria resulted in a final sample size of n=1577 (OSMF-500, lichen planus-500, lichenoid reaction-77, and leukoplakia-500). All included cases were not histopathologically confirmed. We have also included cases that were clinically diagnosed and responded well to pharmacotherapy. Random sampling method was applied for the selection of cases except for lichenoid reactions since the available cases were limited. Further, the selected OPMD cases were evaluated concerning their chief complaints and tabulated as two subsections: self-referral cases and specialist-referral cases. Patients who reported oral mucosal lesions as their primary complaint to the dental college were considered self-referral cases. Patients referred to the dental department for reasons other than lesions of the oral mucosa fell into the category of specialist referrals. Cross-verification of the retrieved data was done by two independent observers. Data was tabulated using Microsoft Excel (Microsoft® Corp., Redmond, WA) software and the tabulated data was then transported to IBM SPSS Statistics for Windows, Version 23 (Released 2015; IBM Corp., Armonk, New York, United States) for statistical analysis. Descriptive frequency analysis was carried out using SPSS for each group of OPMDs. To evaluate the significance of the existing association between referral type, gender, and type of OPMDs, Pearson's chi-square test was used. A p-value less than 0.05 was considered significant and the results were represented using bar diagrams.

## Results

On analyzing the demographic and clinical data from n=1577 samples of OPMDs, Table 1 shows the distribution of cases selected for the present study.

**Table 1:** The number, percentage of OPMD cases, and gender distribution of each group included in the present study. OPMD: oral potentially malignant disorder; OSMF: oral submucous fibrosis

Sample groups	Sample size (n)	Percentage	Male	Female
OSMF	500	31.7%	322	178
Oral lichen planus	500	31.7%	173	327
Lichenoid reaction	77	4.8%	17	60
Leukoplakia	500	31.7%	393	107
Total	1577	100%	905	672

Out of 1577 included cases, 727 cases (46.1%) were histopathologically confirmed and 850 cases (53.9%) were clinically diagnosed. Out of 1577 cases, 960 (60.8%) were associated with various habits like chewing tobacco, arecanut, and smoking. However, 617 cases (39.1%) included in the present study were not associated with any deleterious habits.

Out of 1577 cases of OPMDs, 929 (58.9%) were specialist-referral cases and 648 (41.1%) were self-referral cases. Table 2 shows the total number of cases and distribution of cases based on types of referral, gender, and OPMDs.

**Table 2:** The total number of cases and distribution of cases based on types of referral, gender, and OPMDs. OPMDs: oral potentially malignant disorders; OSMF: oral submucous fibrosis

OPMDs			Types of referral		Total
			Specialist-referral	Self-referral	
OSMF	Gender	Male	157	165	322
		Female	73	105	178
	Total		230	270	500
Lichen planus	Gender	Male	86	87	173
		Female	104	223	327
	Total		190	310	500
Lichenoid reaction	Gender	Male	6	11	17
		Female	33	27	60
	Total		39	38	77
Leukoplakia	Gender	Male	376	17	393
		Female	94	13	107
	Total		470	30	500
Total	Gender	Male	625	280	905
		Female	304	368	672
	Total		929	648	1577

**Figure 1:**
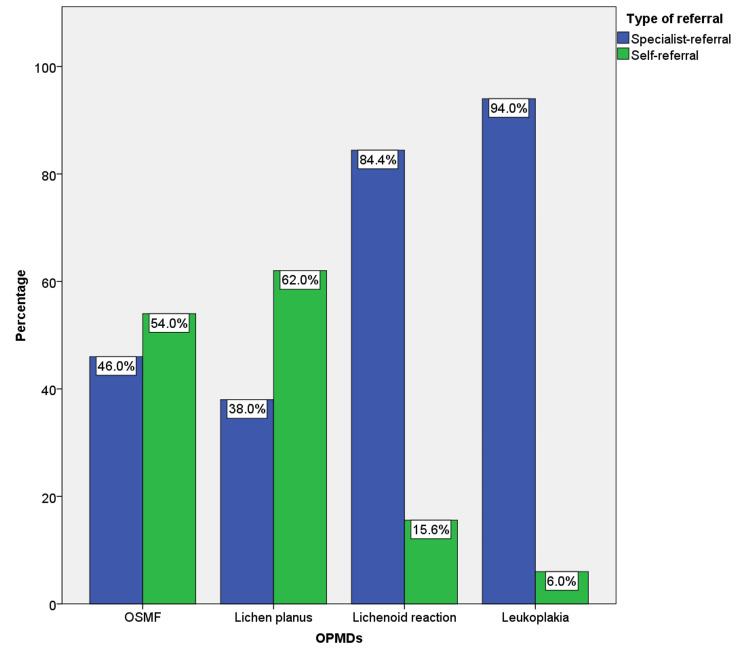
The bar graph shows the association between the type of referral and OPMD cases. OPMD: oral potentially malignant disorder; OSMF: oral submucous fibrosis; X-axis showing OPMD cases grouped as OSMF, lichen planus, lichenoid reaction, and leukoplakia. Y-axis depicts the percentage of cases. Pearson's chi-square test p-value=0.00

In the present study, we found that 310 (62%) lichen planus cases and 270 (54%) OSMF cases were more self-referred when compared to 38 (15.6%) lichenoid reactions and 30 (6%) leukoplakia cases which were more specialist referred. The association between the OPMD type and referral type was determined to be statistically significant with a p-value of 0.00 (Figure 1).

**Figure 2:**
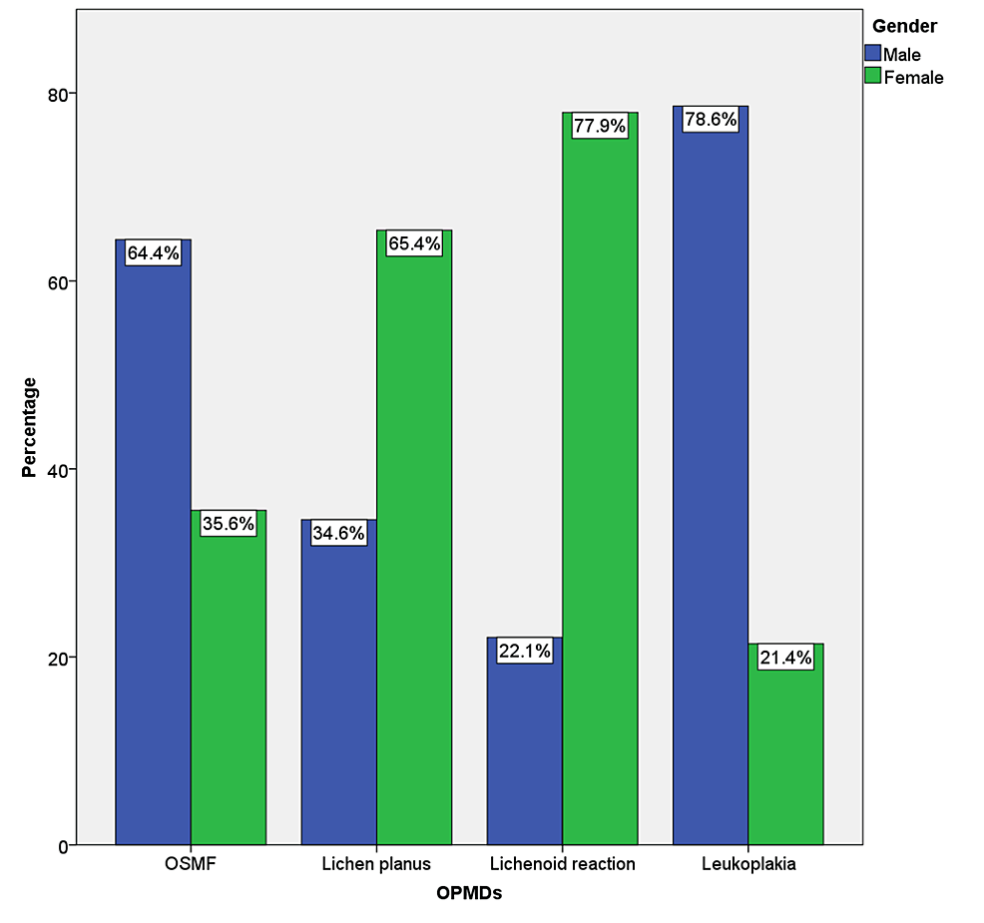
The bar graph shows the distribution of affected males and females in each group of OPMDs included in the present study. OPMDs: oral potentially malignant disorders; OSMF: oral submucous fibrosis

On analyzing the gender predilection, males were found to be predominantly affected in OSMF and leukoplakia groups with 322 (64.4%) and 393 (78.6%), respectively when compared to females. On the contrary, females were more commonly affected in 327 (65.4%) lichen planus and 60 (77.9%) lichenoid reactions. The gender distribution among the selected samples is represented in Figure 2.

**Figure 3:**
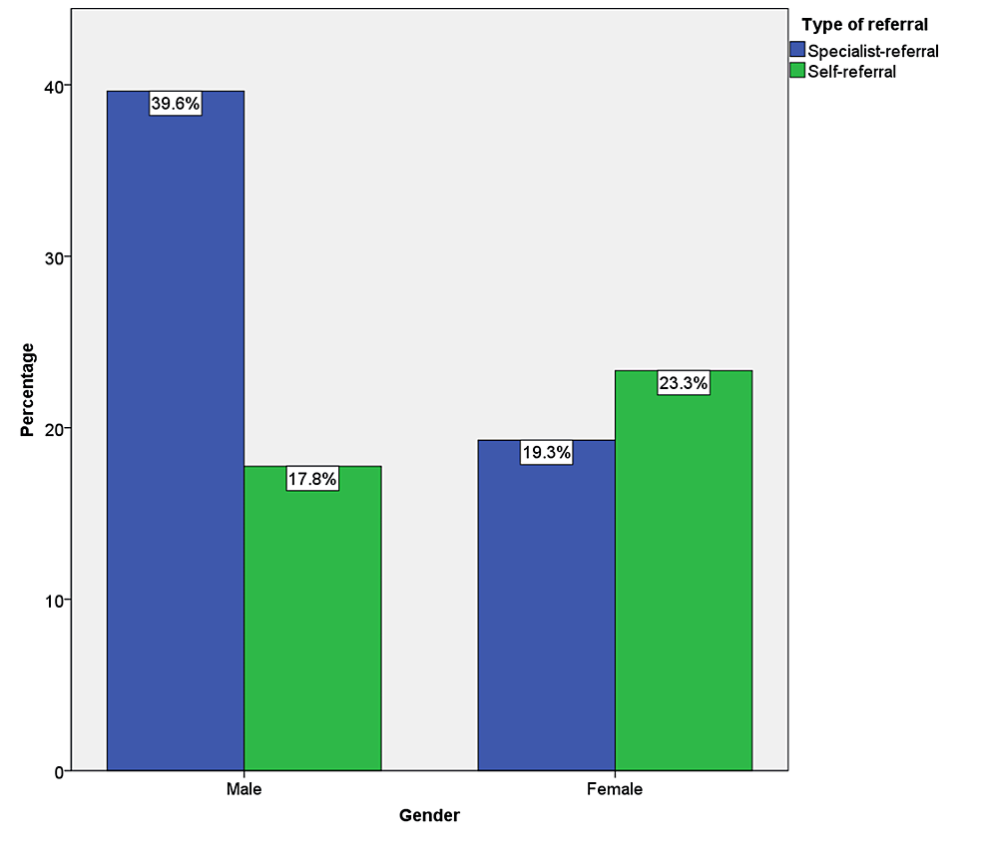
The bar graph depicts the association between gender and type of referral in OPMDs. OPMDs: oral potentially malignant disorders; the X-axis represents the gender and the Y-axis represents the percentage of cases. Self-referred cases were significantly more common in females than males (p=0.00)

When the association between the type of referral and gender was studied, the association was statistically significant with a p-value of 0.00. Among the total sample, 905 (57.4%) were males and 672 (42.6%) were females. Among affected males, 625 (39.6%) were referred by a specialist for the diagnosis of oral mucosal lesions, but only 280 (17.8%) were self-referred. However, in females, only 304 (19.3%) cases were specialist referred but 368 (23.3%) were self-referred (Figure 3).

**Figure 4:**
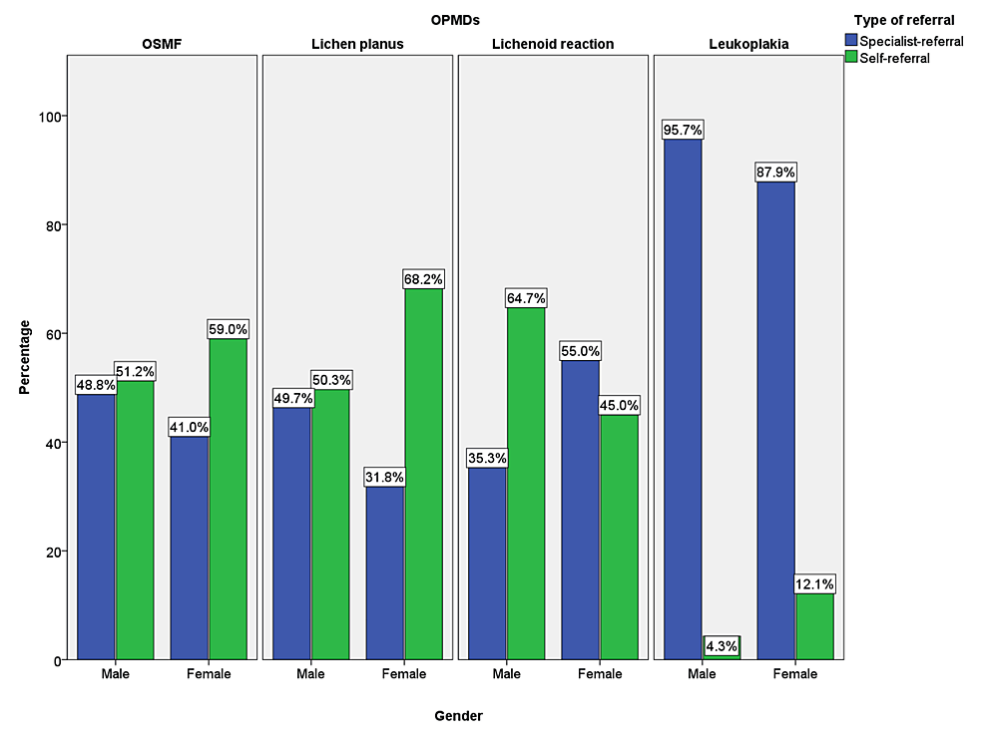
The bar graph depicts the percentage distribution of self-referred and specialist-referred cases among males and females in OSMF, oral lichen planus, lichenoid reactions, and leukoplakia. OSMF: oral submucous fibrosis; OPMDs: oral potentially malignant disorders; self-referred cases are represented by green and specialist-referred cases are represented by blue color. Leukoplakia was the least self-referred lesion compared to lichen planus and OSMF

When the type of referral and gender distribution in OPMDs were cross-tabulated, it was found that lichen planus was more self-referred followed by OSMF and leukoplakia was the least self-referred group. In lichen planus, 223 (68.2%) females were self-referred when compared to 87 (50.3%) males. Similarly, 105 (59%) female patients affected with OSMF were self-referred to that of 165 (51.2%) males affected with OSMF. In leukoplakia, 13 (12.1%) and 17 (4.3%) of the affected females and males were self-referred. On the contrary, 11 (64.7%) self-referred males were more compared to 27(45%) self-referred females in lichenoid reaction (Figure 4).

## Discussion

In 2017, the WHO defined OPMDs as a clinical presentation that carries a risk of cancer development in the oral cavity whether in a clinically definable precursor lesion or a clinically normal mucosa [12]. However, the WHO in 2022 added lichenoid reactions into the OPMD category and deleted chronic candidiasis, syphilitic glossitis, and actinic keratosis [13]. Apart from the most common oral leukoplakia, the other OPMDs are erythroplakia, erythroleukoplakia, OSMF, palatal lesions of reverse cigar smoking, oral lichen planus, and lichenoid reactions [14]. However, we have included leukoplakia, lichen planus, lichenoid reactions, and OSMF in this study, considering the limited availability of cases like erythroplakia. OPMDs can undergo malignant transformation due to persisting etiologies at a varied rate [15]. The presence of epithelial dysplasia on a histological examination, gender, site, lesional factors, and habit-associated factors can contribute to the malignant transformation of OPMDs [7]. It is strongly advised that suspicious lesions can be identified by a specialist as soon as possible to identify any malignant changes present in an early phase thereby reducing the likelihood of aggressive outcomes like malignization [16].

To our knowledge, this was the first preliminary institution-based attempt to evaluate the clinical awareness among the South Indian population about OPMDs like leukoplakia, lichen planus, OSMF, and lichenoid reactions by retrospectively quantifying the type of referral in each reported case during the past five years. In the present study, we found that lichen planus (62%) and OSMF (54%) cases were more self-referred compared to lichenoid reaction (15.6%) and leukoplakia (6%). The variation between groups was found to be statistically significant with a p-value of 0.00. This also reiterated that most of the leukoplakia and lichenoid reaction cases reported to our institution were diagnosed as a part of routine oral examination when the patient reported other chief complaints. This observation highlights the need for awareness among the general population about the clinical presentation of OPMDs, their course of progression, and malignant transformation. Even though the malignant transformation potential of leukoplakia is significantly high, this category was found to be the least self-identified and referred by patients [17]. On the contrary, lichen planus was the most self-referred followed by OSMF. The reason for this could be the more symptomatic presentation of oral lichen planus when compared to leukoplakia. Oral lichen planus is usually presented as a burning sensation during eating spicy foods [18] but conversely, leukoplakia is a white patch or plaque which is usually asymptomatic until the time of its malignant transformation [19]. Self-referrals among the OSMF cases were also significantly high compared to leukoplakia. The difficulty in mouth opening can be a solid reason for the increased self-referral rate among the OSMF cases [20]. Regardless of the similarity of clinical features in lichenoid reactions and lichen planus, only 15.6% of the lichenoid reactions were self-referred compared to 62% of lichen planus. This can be due to the smaller sample size of lichenoid reactions in the present study. Previously published studies also reported less awareness of the general population about OPMDs. A survey conducted by Rai et al. in 2017 found that the awareness of the general population about OPMDs was significantly less when compared to oral cancer [21,22]. Since the previously available data in this domain is mainly based on surveys, it can be biased. The authenticity of results obtained from the actual retrospective patient data analysis was more when compared to surveys. Hence in that aspect, this study gives a clearer picture of the lack of awareness about OPMDs in the current scenario.

In gender predilection analysis, males were found to be predominantly affected in leukoplakia and OSMF; however, females were more commonly affected in lichen planus and lichenoid reactions. This was in concordance with previous studies [23-25]. Leukoplakia and OSMF were mainly habit-associated diseases, hence the male dominance in these two groups can be attributed to the increased prevalence of tobacco and arecanut chewing habits in males [25]. Lichen planus and lichenoid reactions were more common among females, because of the immune-related etiology of these diseases [26]. Even though we have calculated the habit-associated cases in our study, we have not evaluated in detail the association of habit with other parameters, because the present study focused predominantly on the type of referral. However, we are currently working on an extended retrospective analysis of OPMDs with the inclusion of all the demographic, clinical, and etiopathological parameters and follow-up history.

When gender was cross-tabulated with the type of referral, self-referred cases were more observed among females (23.3%) than males (17.8%) (p=0.00). The increased number of self-referred cases in females in the present study could be attributed to the augmented observation power and allotment of more time for self-care in females. Deeks et al. in 2009 stated that compared to men, women were more likely to believe that it was their duty to seek advice on disease prevention [27]. Upon detailed gender analysis, OPMDs except lichenoid reactions showed female patients with more self-referral than males. Even though this was a preliminary study, the study results outspoken the need for awareness among the general population about OPMDs and their malignant transformation potentials. The results of the present study can be considered as a model for more institutional-level studies and can be considered as an aid at the institutional level to improve awareness among patients. To close the gap, awareness campaigns at the institutional level and dental camps stressing the importance of early diagnosis of suspicious oral lesions can be held at different strata of society. This will allow many cases of OPMDs to be treated at an early stage and prevent more serious consequences like malignant transformation.

The present study was conducted on cases with proper diagnosis and data availability. Not all included cases underwent biopsy, because few cases were clinically diagnostic and responsive to pharmacotherapy treatment. Hence, histopathological confirmation was not available for all of the included cases. Other limitations include limited geographical area of sample selection, biases in history taking, errors in data recording, single institutional study design, and lack of inclusion of a detailed habit history.

## Conclusions

This study measured the need for awareness of OPMDs among the general population. Since the symptomatic lesions like lichen planus and OSMF were more self-referred, the importance of identifying the less symptomatic lesions like leukoplakia and its potential for transforming into oral cancer also should be stressed among the general population. The lack of awareness of OPMDs among patients can result in delayed diagnosis and treatment, which may further result in progression to aggressive outcomes. Building awareness at both the Institutional level and public level on OPMDs is the need of the hour. Campaigns and classes can be conducted at panchayat, and corporation levels to reach out to more people. In the absence of such campaigns, the early diagnosis and prevention of oral cancer remain a distant possibility.
